# Diet, Nutrition and Chronic Degenerative Diseases

**DOI:** 10.3390/nu13041372

**Published:** 2021-04-20

**Authors:** Laura Di Renzo, Paola Gualtieri, Antonino De Lorenzo

**Affiliations:** 1 Section of Clinical Nutrition and Nutrigenomic, Department of Biomedicine and Prevention, University of Rome Tor Vergata, Via Montpellier 1, 00133 Rome, Italy; paola.gualtieri@uniroma2.it (P.G.); delorenzo@uniroma2.it (A.D.L.); 2 School of Specialization in Food Science, University of Rome Tor Vergata, 00133 Rome, Italy

Chronic degenerative diseases (CDDs), represented mainly by obesity, cardiovascular disease (CVD), diabetes, chronic kidney disease (CKD), inflammatory bowel diseases, osteoporosis, sarcopenia, neurodegenerative diseases such as Huntington’s disease (HD), rheumatoid arthritis (RA), chronic respiratory diseases, and many cancers, have been, up to now, the most frequent causes of prolonged disability and death worldwide [1]. About 17 million people die prematurely each year from a CDD and the number is expected to grow. Worldwide, patients with one or more CDDs represent over 30% of the population, and 70–80% of public health resources are spent today on the management of CDDs [1]; the data become even more worrying considering the most recent epidemiological projections, according to which in 2030 they will represent 80% of all diseases in the world [2].

According to the Global Burden of Disease, the major determinants of CDDs, in terms of Disability Adjusted Life Y (DALY, which is equal to the sum of years of life lost due to premature death and those experienced in disease rather than health), are mainly attributable to behavioral risk factors such as poor nutrition, reduced fruit and vegetable intake, high body mass index (BMI) (≥25.0 Kg/m^2^), cigarette smoking habit, high alcohol consumption, and low level of physical activity [3]. The main dietary contributing factors to deaths were: (a) reduced consumption of fruit (4.9 million attributable deaths/year), of vegetables (1.8 million), of nuts and seeds (2.5 million), of whole grains (1.7 million); (b) high sodium consumption (3.1 million).

Based on this background, the volume entitled “Diet, Nutrition and Chronic Degenerative Diseases” collects contributions focused on understanding the role of healthy, personalized nutrition, bioactive molecules and microbiota in the prevention and management of CDDs.

A range of original articles in this Special Issue (SI) highlighted the positive effect of the Mediterranean diet (MD), characterized by the extensive use of olive oil, cereals, fruit, vegetables, nuts, legumes, fish, many condiments and spices, the moderate consumption of red wine, and a low amount of dairy and meat, to prevent and manage obesity, CVD, CKD, RA and other CDDs.

Noce et al. highlighted the role of the MD in the treatment of CKD and its comorbidities, putting an effort on the antioxidant and anti-inflammatory effects of extra virgin olive oil (EVOO) [4]. Moreover, Domingues et al. proposed a supplementation protocol with creatine (Cr) to improve functional capacity and muscle oxygen saturation (StO2) in patients with symptomatic peripheral artery disease (PAD) [5]. A randomized controlled study conducted by Jalal et al. [6] proposed the consumption of Phaseolus Vulgaris (PV) to increase the urinary volume and enhance the elimination of small kidney stones.

Quattrini et al. considered the role of MD adherence level and dietary calcium intake for maintaining good bone health and preventing osteoporosis [7].

Di Renzo et al. demonstrated the role of a modified MD (mMeD), a low carbohydrate diet enriched with antioxidant compounds, as a strategy for lipoedema treatment, able to reduce the subcutaneous limbic adipose tissue that characterizes this disease and improve quality of life, such as by decreasing pain, anxiety and discomfort [8].

Lombardo et al. [9] showed the efficacy of MD, associated with aerobic physical activity, to promote weight loss in postmenopausal women and to reduce the cardiovascular and metabolic risk factors. Moreover, in obese subjects, MD adherence, assessed by PREDIMED (Prevención con Dieta Mediterránea) score, improves sleep quality and shifts the evening chronotype, associated with shorter sleep duration, later sleep timing, and caloric consumption after 8:00 p.m., towards the morning chronotype, associated with consuming more healthy foods, such as vegetables, fruits and fish, as proved by Moscogiuri et al. [10,11].

An interesting review by Merra et al. [12] summarizes the important role of polyphenols, polyunsaturated fatty acids (PUFA) ω-3 and fiber in high quantity in the typical food of the MD to control eubiosis. Moreover, since gut dysbiosis has been involved in the onset of autoimmune diseases such as RA, MD is recommended for RA patients to reduce the inflammatory mediators and a percentage of favored phyla, such as Bacteroidetes and the Firmicutes/Bacteroidetes ratio [12].

To reduce the CVD risk, Macarro et al. proposed the consumption of active products of immature citrus fruits, grapefruit (Citrus paradisi Macfad), bitter orange (*Citrus aurantium* L.) and olive leaf (*Olea europaea* L.), capable of improving flow-mediated vasodilation (FMD), blood pressure (BP), lipid profile, thrombotic status, oxidative stress, inflammation, anthropometric variables and quality of life [13]. In addition, a low dietary acid load (DAL) due to a low intake of acidogenic foods (including meat, fish, cheese, rice and cereals) and a high intake of alkaline foods (fruits, vegetables, legumes, potatoes and red wine) contributed to a reduction in cardiometabolic disorders [14]. Therefore, the Western dietary pattern could be associated with the prevalence of cardiometabolic risk factors.

An interesting cross-sectional study on Parkinson’s disease (PD) showed how changes in energy intake could be an important contributing factor to the reduction in involuntary weight loss and disease progression [15].

HD, a rare neurodegenerative disease, has been associated with low MD adherence. Therefore, increasing Mediterranean food intake and supplementing with specific nutrients, such as triheptanoin, L-acetyl-carnitine and creatine, could be the way to improve the course of HD [16].

On the basis of the papers published in the SI, it emerges that the nutritional transition of recent decades, characterized by the transition from the consumption of foods based on cereals, fruit and vegetables to foods rich in saturated fats (mainly meat and dairy products) and simple sugars, associated with a diet characterized by a higher energy intake, represents the main risk factor in the onset of CDDs.

As suggested in the SI, a solution to arrest the increase in CDDs, at least in part, could be guaranteed by preventive interventions in lifestyles and eating behaviors. The Mediterranean diet and healthy and sustainable dietary pattern promotion are aimed at reducing the modifiable exogenous risk factors, associated with the risk of obesity, CVD, CKD, RA and other CDDs.

## Data Availability

Not applicable.
